# Protective Role of Lipid-Lowering Drugs in Breast Cancer: Effects on Cancer Incidence and Cardiotoxicity

**DOI:** 10.3390/life15111749

**Published:** 2025-11-14

**Authors:** Alfredo Mauriello, Adriana Correra, Anna Chiara Maratea, Celeste Fonderico, Arianna Amata, Valeria Cetoretta, Vincenzo Russo, Antonello D’Andrea

**Affiliations:** 1 S.C. Cardiology, Institute National Cancer, IRCCS, Foundation “G. Pascale”, Via M. Semmola 52, 80131 Naples, Italy; alfredo.mauriello93@libero.it (A.M.); celeste.fonderico@istitutotumori.na.it (C.F.); 2 Cardiology Department, Ospedali Riuniti University Hospital, Viale Pinto 1, 71122 Foggia, Italy; adrianacorrera@gmail.com; 3 Department of Cardiovascular Disease, ASL Napoli 1 Centro, Via Comunale del Principe, 13a, 80145 Napoli, Italy; annachiara.maratea@gmail.com; 4 Department of Cardiovascular Medicine, Fondazione Policlinico Universitario A. Gemelli, IRCCS, L.go A. Gemelli 8, 00168 Rome, Italy; arianna.amata2@gmail.com; 5 Cardiology and Arrhythmology Clinic, University Hospital “Ospedali Riuniti”, Marche Polytechnic University, Via Conca 71, 60126 Ancona, Italy; vcetoretta@gmail.com; 6 Cardiology Unit, Department of Medical and Translational Sciences, Monaldi Hospital, University of Campania “Luigi Vanvitelli”, Via L. Bianchi snc, 80131 Naples, Italy; vincenzo.russo@unicampania.it; 7 Cardiology and Intensive Care Unit, Department of Cardiology, Umberto I Hospital, Via A. de Nicola 1, 84014 Nocera Inferiore, Italy

**Keywords:** statins, chemotherapy, cardiotoxicity, cancer prevention, cardio-oncology, lipid-lowering drugs, PCSK9

## Abstract

The bidirectional link between cancer and cardiovascular disease presents a growing clinical challenge, often exacerbated by the cardiotoxic effects of many anti-cancer drugs. Simultaneously, the incidence of cardiovascular pathologies in cancer patients is on the rise. Recently, lipid-lowering drugs, particularly statins, have emerged not only as first-line cardiovascular drugs but also as potential agents with pleiotropic effects that could influence cancer progression and mitigate cardiotoxic side effects. We will analyze data from epidemiological studies, clinical trials, and laboratory research. This comprehensive review aims to examine and synthesize the current evidence regarding the use of lipid-lowering drugs in breast cancer patients, focusing on two main areas: their potential protective role in reducing the incidence of breast cancer and their impact on the prevention and management of treatment-induced cardiotoxicity.

## 1. Introduction

The growing number of cancer survivors has led to increased attention to the long-term sequelae of oncological treatment, particularly cardiovascular complications, which represent a significant source of morbidity and mortality [1]. In parallel, there has been a rising interest in the repurposing of lipid-lowering drugs, traditionally used for the prevention of atherosclerotic cardiovascular diseases (ASCVD) [2,3], for their pleiotropic effects that could influence both cancer progression and chemotherapy-related cardiotoxicity (CTRCD) [4]. Beyond their cholesterol-lowering effect, statins exert pleiotropic properties, including anti-inflammatory and antiproliferative effects [5,6]. These effects can attenuate the inflammatory response, inhibit tumor cell proliferation, and reduce cancer progression and micrometastasis [7,8].

The use of lipid-lowering drugs in oncological patients is focused on two main areas: the potential for chemoprevention or improvement of cancer prognosis, and protection against CTRCD. While preclinical investigations into the application of lipid-lowering drugs in the field of cardio-oncology are emerging [9], a critical need remains for dedicated clinical trials, especially those focusing on novel agents such as anti- proprotein convertase subtilisin/kexin type 9 (PCSK9) drugs. Cardiotoxicity related to drugs like anthracyclines and anti-HER2 agents is a significant clinical challenge that can lead to cardiac dysfunction and heart failure [10,11].

**Figure 1:**
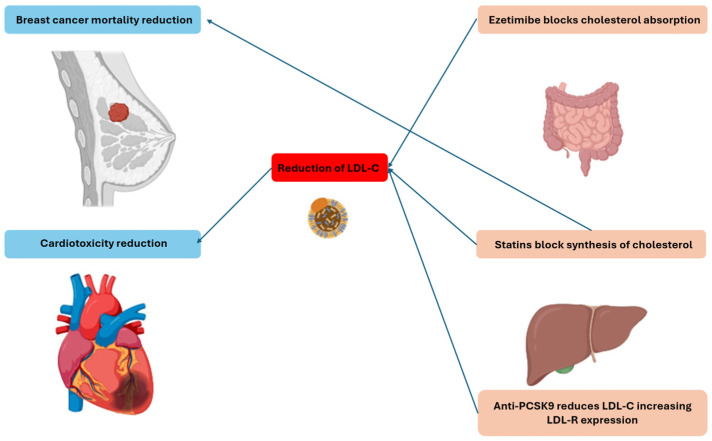
Relationship between lipid-lowering drugs and breast cancer mortality and cardiotoxicity. LDL-C: low-density-lipoprotein circulant; LDL-R: low-density-lipoprotein receptor; PCSK9: proprotein convertase subtilisin/kexin type 9.

This narrative review examines the actual scientific evidence on the use of lipid-lowering drugs, particularly statins, ezetimibe, and PCSK9 inhibitors, in breast cancer patients. The review focuses on the dual action of these agents: the potential reduction of breast cancer incidence, recurrence, and mortality and the cardioprotective effects against toxicity induced by therapies such as anthracyclines, trastuzumab, and radiotherapy. Figure 1 illustrates the direct and indirect effects of lipid-lowering drugs on reducing breast cancer mortality and cardiotoxicity.

## 2. Material and Methods

This article is structured as a narrative, clinically oriented review regarding the state of the art about the relationship between the use of lipid-lowering drugs and breast cancer. Its primary aim is to establish the pathophysiological link between use of lipid-lowering drugs and breast cancer and to explore potential preventive and therapeutic strategies in breast cancer patients with a rational synthesis of evidence, avoiding the production of a formal systematic review or meta-analysis.

To guarantee authoritativeness and transparency in the selection of sources, we used a search strategy that guided, but did not solely determine, the narrative synthesis. A comprehensive search was conducted in PubMed/MEDLINE, EMBASE and Web of Sciences for articles published from January 2015 to October 2025, employing juxtaposition of the following keywords and Boolean operators: “breast cancer” OR “cancer” AND (“lipid-lowering drugs” OR “statin” OR “ezetimibe” OR “PCSK9” OR “fibrates” OR “lipid”). The search was limited to English-language documents with visible abstracts.

Notably, we included prospective and retrospective clinical studies, randomized controlled trials, meta-analyses, and registry reports addressing the relationship between the use of lipid-lowering drugs and the risk of breast cancer and the incidence of risk of cardiotoxicity in breast cancer patients.

We excluded case reports, editorials, commentaries, comprehensive reviews, experimental pre-clinical studies without clear clinical implications, and conference abstracts without clinical complete data.

This review is not systematic, so no aggregate quantitative synthesis was performed. However, we assessed the quality of the included evidence by considering key domains relevant to clinical trials: patient selection (representativeness of study populations, inclusion/exclusion criteria); outcome ascertainment and follow-up (definition and measurement of cardiotoxicity or clinical endpoints); and control for confounding (adjustment for baseline cardiovascular risk factors, treatment exposure, and competing risks). For observational studies, we performed a qualitative appraisal based on the Joanna Briggs Institute (JBI) Critical Appraisal Checklists [12] and, when applicable, by the Cochrane Risk-of-Bias framework for randomized trials. The use of these aspects was used to define the relative weight assigned to different types of evidence in the discussion.

The search strategy we used was reproducible to ensure transparency. However, due to the heterogeneity of the study designs, which spanned everything from clinical trials and registries to models of organization and policy analyses, we could not follow the complete PRISMA guidelines. Specifically, we omitted steps like screening for duplicates, formally assessing the risk of bias, and performing a meta-analysis, because our main objective was the practical implementation and management of health systems. We determined that a PRISMA-based systematic review would be inappropriate for this work. To emphasize this methodological difference, we state explicitly that we performed a structured narrative synthesis based on the search results, rather than a systematic review. Throughout the text, we have made an effort to indicate the strength of the evidence, noting if conclusions originated from meta-analyses, extensive multicenter studies, or smaller observational series, so the reader can assess the credibility of the findings.

## 3. Molecular Mechanisms in Cancer Incidence Reduction

Most of the lipid-lowering drugs studied for anticancer activity, such as statins, act primarily on the mevalonate (MVA) pathway [13].

### 3.1. Statins

Statins are candidate drugs for repurposing in cancer treatment. Their primary mechanism is the inhibition of 3-hydroxy-3-methylglutaryl-CoA (HMG-CoA) reductase, the rate-limiting enzyme in the mevalonate pathway. The anticancer molecular mechanisms include:

#### 3.1.1. Inhibition of Isoprenoids and Oncogenic Pathways

**Figure 2:**
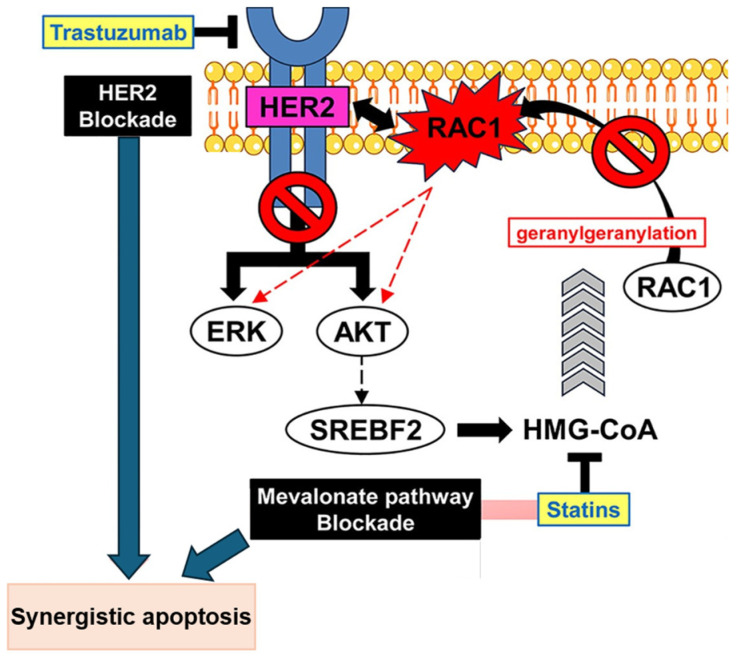
Illustration of synergistic action of trastuzumab and statins in induce apoptosis. In human epidermal growth factor receptor (HER)-2 positive breast cancer cells, the combination of statins with anti-HER2 agents induces apoptosis by inhibiting protein kinase B (AKT) and extracellular signal-regulated kinases phosphorylation. This effect is mediated by the inhibition of geranylgeranylation, which restores the dephosphorylation of AKT and ERK. The geranylgeranylated protein Ras-related C3 botulinum toxin substrate 1 (Rac1) indirectly activates the AKT and ERK pathways. AKT: protein kinase B; EKR: extracellular signal-regulated kinases; HER2: human epidermal growth factor receptor 2; HMG-CoA: β-Hydroxy β-methylglutaryl-CoA; Rac1: Ras-related C3 botulinum toxin substrate 1; SREBP-2: sterol regulatory element-binding protein 2.

The MVA pathway is essential for the synthesis of cholesterol and isoprenoids, such as farnesyl diphosphate (FPP) and geranylgeranyl pyrophosphate (GGPP). Statins deplete intracell reserves of isoprenoids, which are important regulators of tumor cell proliferation and metastasis [14]. The inhibition of geranylgeranylation plays an important role in antitumoral activity, as it is involved in activating oncogenic kinase signaling [7]. In human epidermal growth factor receptor (HER)-2 positive breast cancer cells, the combination of statins with anti-HER2 agents induces apoptosis by inhibiting AKT and extracellular signal-regulated kinases (ERK) phosphorylation. This effect is mediated by the inhibition of geranylgeranylation, which restores the dephosphorylation of AKT and ERK [7]. The geranylgeranylated protein Rac1 indirectly activates the AKT and ERK pathways. Statins block the membrane localization of the geranylgeranylated Rac1 protein [7]. Figure 2 represents this mechanism.

#### 3.1.2. Induction of Apoptosis and Ferroptosis

Combined treatment with statins and anti-HER2 agents significantly increases Caspase 3/7 activity, indicating the induction of apoptosis. In Triple-Negative Breast Cancer (TNBC) cells, simvastatin (SIM) can induce ferroptosis. This occurs because SIM inhibits HMG-CoA reductase expression, downregulating the MVA pathway and glutathione peroxidase 4 (GPX4), an enzyme that prevents cellular oxidative damage [15].

#### 3.1.3. Immune Modulation and Anti-Inflammation

Inhibition of the HMG-CoA reductase target is associated with a reduced risk of breast cancer. This suggests that part of statins’ action may occur through pathways not directly related to lipid lowering, such as modulating immune function and reducing inflammation. Statins can enhance the anti-tumor immune response by reducing cluster of differentiation (CD)8+ T cell exhaustion and suppressing the expression of programmed death-ligand 1 (PD-L1) [16].

### 3.2. Ezetimibe

Ezetimibe inhibits the absorption of cholesterol through the Niemann-Pick C1-Like 1 (NPC1L1) receptor [17]. It has shown anticancer mechanisms that include anti-angiogenesis effect by inhibiting neovascular growth, cell proliferation, and promoting apoptosis. Therefore, ezetimibe seems to have anti-inflammatory effects through promoting anti-cancer immunity by enhancing the memory function and infiltration of CD8+ T lymphocytes and reducing mTORC2 signaling in these lymphocytes [18].

## 4. Molecular Mechanisms in Cardiotoxicity Reduction

Lipid-lowering drugs, particularly statins and PCSK9 inhibitors, can be used to mitigate chemotherapy-induced cardiotoxicity [19].

### 4.1. Statins

Statins exert a cardioprotective effect through their pleiotropic properties:

#### 4.1.1. Anti-Inflammatory and Antioxidant Effects

Statins determine anti-inflammatory and antioxidant benefits that improve maladaptive cardiac remodeling and myocardial fibrosis [20]. Some evidence suggests that rosuvastatin has antioxidant and anti-inflammatory effects that may help prevent deoxyribonucleic acid (DNA) damage and provide a cardioprotective effect [21]. Mechanisms include the ability of statins to reduce the gene expression of myeloperoxidase (MPO), a marker of inflammation and cardiotoxicity. Rosuvastatin, in randomized controlled trial, has been shown to reduce the levels of interleukin (IL)-6 and high-sensitivity cardiac troponin I (hs-cTnI) in 50 newly diagnosed HER2-positive breast cancer patients and chemotherapy-treated patients [22,23].

#### 4.1.2. Inhibition of Fibrosis and Cardiomyocyte Protection

Preclinical studies suggest that statins preserve systolic function and calcium management and reduce oxidative stress and myocardial cell death during anthracycline exposure [24,25,26]. In a subgroup including 171 participants aged 52 years (53% men) in the Statins to Prevent the Cardiotoxicity From Anthracyclines (STOP-CA) trial [26], atorvastatin significantly reduced the likelihood of an increase in myocardial extracellular volume (ECV) in patients with regimen of anthracycline-based chemotherapy (*p* < 0.001) [27]. Statins inhibit Rho GTPases, reducing the production of reactive oxygen species (ROS) and limiting topoisomerase II-mediated DNA damage. These damages are caused by anthracycline-induced cardiotoxicity.

#### 4.1.3. Protection Against Immune Checkpoint Inhibitor (ICI) Cardiotoxicity

High-dose atorvastatin has been shown to mitigate functional deficits by inhibiting coronary microvascular endothelial dysfunction, a phenomenon that precedes cardiotoxicity induced by pembrolizumab [28]. Therefore, atorvastatin prevented endothelial toxicity, decreasing endothelial activation markers like intercellular adhesion molecule 1 (ICAM-1) and vascular adhesion molecule 1 (VCAM-1) induced by pembrolizumab in in vitro models [28].

### 4.2. PCSK9 Inhibitors

PCSK9 inhibitors, including inclisiran, a silencing ribonucleic acid [29], and alirocumab [30] and evolocumab [31], two monoclonal antibodies, are known for their powerful lipid-lowering capabilities and demonstrate significant pleiotropic benefits [32].

#### 4.2.1. Modulation of Inflammation and Oxidative Stress

Inclisiran attenuates doxorubicin (DOXO)- and trastuzumab-induced cardiotoxicity by reducing NLRP3 inflammasome activation and MyD88 signaling [9]. These pathways are essential mediators of cardiac inflammation. PCSK9i have been shown to significantly reduce intracellular ROS and lipid peroxidation products, malondialdehyde (MDA) and 4-hydroxy-2-nonenal (4-HNA) induced by combined chemotherapy [9]. Therefore, PCSK9 inhibition suppresses the upregulation of pro-inflammatory cytokines such as IL-1a, IL-1b, IL-6, TNF-α, IL-12, and IL-17A, and increases the anti-inflammatory cytokine IL-10 [9].

#### 4.2.2. Structural Protection and Apoptosis Prevention

Inclisiran exerts cardioprotective effects by improving cell viability and reducing apoptotic activation, decreasing in caspase-3 activity [9]. Furthermore, PCSK9is preserve mitochondrial integrity restoring mitofusin-2 levels and calcium homeostasis [9]. PCSK9 inhibition also protects against cytoskeleton disruption, preserving F-actin expression induced by sequential DOXO-trastuzumab therapy [9].

#### 4.2.3. PCSK9 and Nuclear Transport

DOXO-induced cardiotoxicity upregulates PCSK9 expression in myocardial tissue [33]. The suggested mechanism is that DOXO-induced PCSK9 accumulates in the cardiomyocyte nucleus, where it interacts with the nuclear transport protein importin subunit beta-1 (KPNB1). This interaction is believed to influence the expression of apoptotic genes, such as Bax/Bcl-2 and cleaved Caspase-3/Pro-Caspase-3, thereby promoting cardiomyocyte apoptosis [33]. PCSK9 inhibitors downregulate the expression of these apoptotic proteins and the nuclear accumulation of PCSK9 [33].

## 5. Protective Effects of Lipid-Lowering Drugs in Reducing Breast Cancer Incidence and Progression and Protection Against Cardiotoxicity

### 5.1. Statins

#### 5.1.1. Protective Effects on Cancer Incidence and Progression

##### Preclinical Studies

Statins have been shown to suppress growth and induce apoptosis in tumor cells. Lovastatin inhibits cancer growth and lung metastasis in a murine breast carcinoma model, through a mitochondrial-mediated, p53-independent apoptotic mechanism [34].

Simvastatin exhibited greater cytotoxicity against MDA-MB-231 cells, a type of triple-negative breast cancer model, than MCF-7 cells, a type of normal breast cancer, due to higher HMG-CoA-reductase expression. Simvastatin induces ferroptosis in TNBC cells by inhibiting HMG-CoA reductase, downregulating the MVA pathway, and deactivating GPX4 [35]. While simvastatin demonstrated effects on MDA-MB-231 and MCF-7 breast cancer cells, there is a clear gap between these in vitro findings and clinical applicability, given that the concentrations used were at least one order of magnitude higher than human therapeutic doses.

Statins block the membrane expression of the geranylgeranylated protein Rac1 in HER2-positive BC cells. Blocking HMG-CoA-reductase with statins enhances the efficacy of trastuzumab by modulating the phosphorylation status of PAK [7].

Simvastatin appears to radiosensitize stem cells and differentiated breast cancer cells. Additionally, simvastatin appears to reduce local inflammation caused by post-mastectomy radiation therapy [36].

In vitro and in vivo studies with simvastatin have demonstrated the induction of pyroptosis through activation of the ROS/caspase-1/GSDMD pathway in colon cancer cells, suggesting that statins may act as potential therapeutic agents for this malignancy. Simvastatin has also been shown to suppress cell proliferation in vitro [37].

##### Clinical Studies

The association between statin use and cancer outcomes has been the subject of numerous clinical studies and meta-analyses [38,39,40,41,42].

Scott et al. [38], in their meta-analysis including forty-one studies, concluded that there was a significant protective association between statin use and both breast cancer death (21 studies, hazard ratio (HR) = 0.81, 95% confidence interval (CI): 0.75–0.87; *p*  <  0.001) and breast cancer recurrence (20 studies, HR = 0.81, 95% CI: 0.74–0.89, *p*  <  0.001). In a cohort of elderly cancer survivors, the use of lipophilic statins was significantly associated with a lower risk of all-cause and cancer-related mortality (HR  =  0.75, 95% CI: 0.62–0.91, *p*  =  0.003).

In the Breast International Group (BIG) 1–98 trial [43], including 6193 postmenopausal women with hormone receptor–positive early breast cancer, the use of cholesterol-lowering drugs, including statins, was associated with improved outcomes in postmenopausal patients with early-stage hormone receptor-positive breast cancer (HR 0.79 for disease-free survival). The benefit appears to be more pronounced in ER+ patients [41].

On the other hand, a post hoc multivariate analysis in the Adjuvant Pertuzumab and Trastuzumab in Early HER2-Positive Breast Cancer (APHINITY) Trial [40], including 4804 patients with a median follow-up of 6.2 years, showed that statin use was not associated with invasive disease-free survival (IDFS) (HR, 1.11; 95% CI, 0.80–1.52), distant relapse-free interval (DRFI) (HR, 1.21; 95% CI, 0.81–1.81), or overall survival (OS) (HR, 1.16; 95% CI, 0.78–1.73).

#### 5.1.2. Protective Effects Against Cardiotoxicity

##### Preclinical Studies and Mechanisms

Statins offer anti-inflammatory and antioxidant benefits that are important in mitigating chemotherapy-induced cardiotoxicity, particularly that caused by anthracyclines. Statins inhibit Rho guanosine triphosphate (GTP)-ases, reducing the production of ROS and limiting topoisomerase II-mediated DNA damage, both contributing factors to anthracycline-induced cardiotoxicity [44]. Therefore, atorvastatin protects cardiomyocytes from DOXO toxicity by modulating survivin expression through the inhibition of Forkhead box protein O1 (FOXO1) [45].

In murine models, rosuvastatin has shown potential for modulating oxidative stress and alleviating DOXO-induced cardiotoxicity. It has also been shown to target pro-inflammatory cytokines and oxidative stress to mitigate trastuzumab cardiotoxicity in rats [46].

Atorvastatin demonstrated a prophylactic role against pembrolizumab-induced cardiotoxicity in murine models. Coronary microvascular endothelial dysfunction precedes pembrolizumab-induced cardiotoxicity in vivo, and atorvastatin mitigates functional deficits by inhibiting this dysfunction. Atorvastatin prevented immune-related cytotoxicity in human endothelial cells [27].

##### Clinical Studies

Statin prophylaxis for CTRCD associated with anthracyclines has received attention, although with mixed results in randomized controlled trials.

Despite initially discordant results from some RCTs, statins have received a Class IIa recommendation in the 2022 European Society of Cardiology (ESC) guidelines for patients at high or very high risk of developing cancer therapy-related cardiovascular toxicity [10].

The STOP-CA [47], a multicenter, randomized, double-blind trial compared atorvastatin (40 mg/day) with placebo in 300 lymphoma patients treated with anthracycline-based chemotherapy. The study showed a significant reduction in the primary endpoint (absolute decline of ≥10% in left ventricular ejection fraction (LVEF) to a final value of <55% at 12 months) in the atorvastatin group compared to the placebo group (9% vs. 22%; *p* = 0.002).

A secondary study of the STOP-CA [27], using cardiac magnetic resonance (CMR), provided a mechanistic insight. Atorvastatin significantly reduced the odds of a clinically relevant increase in myocardial extracellular volume (ECV), a marker for expanded myocardial interstitial fibrosis, after anthracycline-based chemotherapy. The odds of a ≥3% increase in ECV were reduced by 80% in the atorvastatin group compared to the placebo group (OR: 0.20; *p* = 0.002). ECV expansion was associated with a clinically relevant decrease in LVEF.

Nabati et al. [48] in their single-blind RCT, including 89 women with newly diagnosed breast cancer, showed that rosuvastatin (20 mg) given 24 h before the first cycle of DOXO and continued for 6 months in HER2-positive breast cancer patients also treated with trastuzumab reduced the decline in LVEF, levels of Hs-cTnI and IL-6 at 3 and 6 months, and MPO at 6 months (intergroup *p* = 0.012).

On the other hand, clinical studies such as PREVENT [49], including 279 patients treated with anthracyclines predominantly for breast cancer (85%) and a smaller cohort of lymphoma patients (15%), and Statins for the primary prevention of heart failure in patients with cancer receiving anthracycline-based chemotherapy (SPARE-HF) [50], including 112 patients, also using atorvastatin (40 mg/day) in patients with solid tumors (mainly breast cancer, 65%) and hematologic malignancies, found no statistically significant difference in CTRCD (LVEF decline ≥ 10%) compared to placebo. The discrepancy in results may be due to differences in populations, chemotherapy regimens, and endpoints.

Statin therapy, particularly rosuvastatin, in a retrospective study [9] including 1471 patients, demonstrated a reduction in the risk of major adverse cardiovascular events (MACE) in breast cancer patients undergoing adjuvant radiotherapy (adjusted HR, 0.34 [95% CI, 0.25–0.44]). Hydrophilic statins, particularly rosuvastatin and pravastatin, demonstrated the most significant dose-dependent risk reduction. Indeed, increasing daily doses were associated with a lower risk of MACE over 5 years compared to statin non-users (12.24% versus 31.70%).

### 5.2. Ezetimibe

#### 5.2.1. Protective Effects on Breast Cancer Incidence and Progression

##### Preclinical Studies and Mechanisms

Ezetimibe has shown potential for treating and preventing various types of cancer. Ezetimibe inhibits angiogenesis, apoptosis, proliferation, inflammation, stem cell inhibition and enhances the immune response. For example, ezetimibe reduces hepatic cholesterol levels and prevents steatosis-induced liver fibrosis and tumorigenesis. In a hypercholesterolemia murine model, it reduced breast tumor growth accelerated by hypercholesterolemia itself. Ezetimibe promotes anti-tumor immunity and attenuates prostate tumor growth and metastasis by suppressing Akt phosphorylation [51].

##### Clinical Studies

The clinical history of ezetimibe in oncology is initially linked to a controversy [52,53,54,55].

The Simvastatin Ezetimibe in Aortic Stenosis (SEAS) trial [52], a randomized, double-blind trial involving 1873 patients with mild-to-moderate, asymptomatic aortic stenosis, during a follow up of 4 years, had as primary outcome a composite of MACE, including death from cardiovascular causes, aortic-valve replacement, nonfatal myocardial infarction, hospitalization for unstable angina pectoris, heart failure, coronary-artery bypass grafting, percutaneous coronary intervention, and nonhemorrhagic stroke. The study suggested a possible increase in cancer incidence in the simvastatin–ezetimibe group (105 vs. 70, *p* = 0.01) and mortality (HR, 1.00; 95% CI, 0.84 to 1.18; *p* = 0.97).

Subsequent large-scale studies, such as the Study of Heart and Renal Protection (SHARP) [53] including 4650 patients, and IMPROVE-IT [54], including 18,144 patients, did not provide credible evidence of an increased cancer risk related to ezetimibe, used alone or in combination with simvastatin (respectively, *p* = 0.89 and *p* = 0.68). The prospective analysis of the IMPROVE-IT trial showed no effect on the incidence of malignancy [55].

Data regarding breast cancer are not available.

### 5.3. PCSK9 Inhibitors

#### Preclinical Studies

Despite the lack of published clinical studies on PCSK9 inhibition during chemotherapy in humans, the preclinical data are robust and indicate a mechanism of action independent of lowering lipids.

DOXO treatment increases the expression of the PCSK9 protein in myocardial tissue and induces its nuclear aggregation in H9C2 cardiomyocytes, suggesting its role in DOXO-induced cardiotoxicity [33].

Evolocumab improved cardiac function, myocardial damage, and fibrosis in DOXO-treated mice, showing a protective effect against DOXO-induced cardiotoxicity. The mechanism involves the reduction of PCSK9 expression and cardiomyocyte apoptosis, reducing in the Bax/Bcl-2 and Cleaved Caspase-3/Pro Caspase-3 ratios [56].

Inclisiran attenuates cardiotoxicity induced by sequential exposure to DOXO and trastuzumab in induced pluripotent stem cell-derived cardiomyocytes (hiPSC-CMs) [9]. Inclisiran significantly improved cell viability and reduced apoptotic activity. It significantly reduced cardiac damage and heart failure biomarkers, including heart-type fatty acid binding protein (H-FABP), troponin T and brain natriuretic peptide (BNP) [9]. Furthermore, inclisiran significantly reduces the intracellular expression of NLRP3 and MyD88, key components in inflammatory pathways involved in cardiotoxicity. This suggests a lipid-independent cardioprotective effect [9].

There are no available data regarding other lipid-lowering drugs. 

## 6. Drug Interactions and Contraindications of Lipid-Lowering Drugs in Cancer Patients

The main limitations to statin use in cancer patients are the significant risk of drug–drug interactions (DDIs) with oncological therapies and the potential for hepatic dysfunction, either pre-existing or treatment-induced [57].

The primary concern stems from the fact that many statins and several chemotherapy agents, targeted therapies, and hormone therapies are metabolized by the same liver enzymes, primarily the cytochrome P450 (CYP450) system [58].

Many oncological drugs are CYP inhibitors. When taken concurrently, they can slow down the metabolism of the statin, leading to dangerously elevated plasma concentrations of the statin. This significantly increases the risk of statin-related side effects, particularly myopathy and, less commonly, hepatotoxicity. Conversely, a statin might affect the metabolism of an anticancer drug, potentially reducing its effectiveness or increasing its toxicity. To mitigate this risk, clinicians often prefer statins with minimal CYP interaction, such as pravastatin or rosuvastatin. Close therapeutic drug monitoring and dose adjustments are essential when concurrent use is unavoidable [59].

Statins carry a low risk of causing dose-dependent and reversible elevations in liver enzymes [60]. This risk becomes a major limitation in cancer patients due to several factors. Most chemotherapy and many newer targeted/immuno-therapies are themselves hepatotoxic. Adding a statin can potentially lead to cumulative liver injury, making it difficult to determine the source of transaminase elevation and potentially necessitating the interruption of essential cancer treatment. Cancer patients may already have compromised liver function due to liver metastases or underlying chronic conditions. Statins are generally contraindicated or require extreme caution in cases of acute liver disease, persistent and significant elevations of transaminases, or decompensated cirrhosis [61].

## 7. Future Perspectives

Despite the accumulation of preclinical and observational evidence supporting the repurposing of lipid-lowering drugs, particularly statins, generalized clinical implementation requires more robust data from randomized trials across heterogeneous populations.

The MAmmary cancer STatins in ER positive breast cancer (MASTER) trial [62], a phase III, randomized, placebo-controlled trial, is currently ongoing to assess whether adding atorvastatin (80 mg/day) to standard therapy improves IDFS in patients with ER-positive breast cancer. The results of this study will be essential in establishing whether statins can be integrated into oncology guidelines as a standard adjuvant therapy.

It is vital to understand the lipid-lowering independent anticancer mechanisms better. For example, in triple negative breast cancer, simvastatin induces ferroptosis, while in colon cancer, it induces pyroptosis. Future research should identify which patient subgroups benefit the most from the pleiotropic effects of statins (e.g., ER+ patients). The potential of statins to modulate inflammation and immune function (as suggested by MR studies on BC and ICI/Pembrolizumab models) requires further investigation.

Despite highly encouraging preclinical findings demonstrating that inclisiran and evolocumab can attenuate CTRCD (DOXO/trastuzumab) through NLRP3/MyD88 inhibition and reduced oxidative stress, independent of lipids, there’s a lack of clinical studies on PCSK9 inhibition in chemotherapy patients. Prospective RCTs are urgently needed to evaluate the long-term impact of these agents on cardiac outcomes and their oncological safety.

The optimal dosage and timing for initiating cardioprotective therapy (before, during, or after chemotherapy or radiotherapy) are not yet fully defined. For atorvastatin, only the high dose has been studied for cardioprotection. Future studies should also explore the potential for synergy with other cardioprotective agents (such as beta-blockers or SGLT2i).

The conflicting evidence regarding metastatic colorectal cancer treated with regorafenib and the potential protective role in ICI toxicity suggests the need to study drug–drug interactions with targeted therapies and immunotherapy specifically.

Developing medications with reduced propensity for drug interactions and fewer adverse effects on metabolic clearance systems is a significant challenge, but one that is essential for optimizing care in cancer patients undergoing highly metabolically demanding chemotherapy.

## 8. Conclusions

Statins have shown robust preclinical anticancer effects through the modulation of the mevalonate pathway, and consistent clinical benefits in reducing cancer-related mortality and recurrence, especially for ER-positive breast cancer (ER+). Furthermore, statins have received a Class IIa recommendation in the 2022 ESC guidelines for the prevention of CTRCD in high-risk patients, supported by clinical studies demonstrating a significant reduction in cardiac dysfunction and myocardial fibrosis. PCSK9 inhibitors, inclisiran and evolocumab, although lacking definitive clinical data in cardio-oncology, have shown promising preclinical cardioprotective effects, independent of lowering lipids, through the modulation of oxidative stress and inflammatory pathways, such as NLRP3/MyD88.

Despite the promising data, clinical results are sometimes discordant due to the heterogeneity of study designs and breast cancer populations. It is fundamental that future studies, such as the MASTER trial, provide definitive randomized evidence to establish the optimal integration of these drugs into oncological and cardio-oncological clinical practice.

## Data Availability

No new data were created or analyzed in this study. Data sharing is not applicable to this article.
